# The role of gut microbiota in prostate inflammation and benign prostatic hyperplasia and its therapeutic implications

**DOI:** 10.1016/j.heliyon.2024.e38302

**Published:** 2024-09-21

**Authors:** Jie Chen, Bo Chen, Bin Lin, Yin Huang, Jinze Li, Jin Li, Zeyu Chen, Puze Wang, Biao Ran, Jiahao Yang, Huijian Huang, Liangren Liu, Qiang Wei, Jianzhong Ai, Dehong Cao

**Affiliations:** a Department of Urology, Institute of Urology, West China Hospital, Sichuan University, Chengdu 610041, China; b West China School of Medicine, Sichuan University, Chengdu 610041, China; c West China Hospital, Sichuan University, Jintang Hospital, Chengdu 610041, China; d Department of Urology, Karamay people's Hospital of Xinjiang Uygur Autonomous Region, China

**Keywords:** Medicine, Gut microbiota, Prostate inflammation, Intestinal epithelial permeability, Gut dysbiosis, Short-chain fatty acids (SCFAs), Lipopolysaccharide (LPS), Therapy

## Abstract

**Background:**

The gut microbiota thrives in a complex ecological environment and its dynamic balance is closely related to host health. Recent studies have shown that the occurrence of various diseases including prostate inflammation is related to the dysregulation of the gut microbiome.

**Objective:**

This review focus on the mechanisms by which the gut microbiota induces prostate inflammation and benign prostatic hyperplasia and its therapeutic implications.

**Materials and methods:**

Publications related to gut microbiota, prostate inflammation, and benign prostatic hyperplasia (BPH) until April 2023 were systematically reviewed. The research questions were formulated using the Problem, Intervention, Comparison/Control, and Outcome (PICO) frameworks.

**Results:**

Fifteen articles covering the relationship between the gut microbiota and prostate inflammation/BPH, the mechanisms by which the gut microbiota influences prostate inflammation and BPH, and potential therapeutic approaches targeting the gut microbiota for these conditions were included.

**Conclusion:**

Short-chain fatty acids (SCFAs), which are metabolites of the intestinal microbiota, protect the integrity of the intestinal barrier, regulate immunity, and inhibit inflammation. However, dysregulation of the gut microbiota significantly reduces the SCFA content in feces and impairs the integrity of the gut barrier, leading to the translocation of bacteria and bacterial components such as lipopolysaccharide, mediating the development of prostate inflammation through microbe-associated molecular patterns (MAMPs).

## 1 Introduction

Many microbes in the human body widely inhabit the respiratory tract, urogenital tract, gastrointestinal tract, and oral cavity. The microbes include bacteria, fungi, viruses, archaea, and protozoans. Among them, bacteria account for the majority [1,2]. Several studies have shown that there are approximately 10^14^ species of bacteria in the human gut, belonging to four main phyla, including *Bacteroidetes, Firmicutes, Actinobacteria*, and *Proteobacteria,* with *Bacteroidetes* and *Firmicutes* being the main phyla [3]. Many complex connections exist between the gut microbiota and host health. Changes in the composition of gut microbiota may be influenced by diet [4], geographical location [5], hormones [6], and host age [7]. In addition, the gut microbiota is involved in host nutrient metabolism, development, immunity, and disease production [8].

The prostate is a high-risk site for benign and malignant diseases, and age is a primary risk factor [9,10]. With increasing age, the incidence of prostate diseases, such as prostate cancer (PCa), prostate inflammation, and benign prostatic hyperplasia (BPH), is also increasing. Acute and chronic inflammatory cell infiltration is often observed in male prostate biopsies. Previous studies have shown that 35%–100 % of prostate biopsies show histological evidence of inflammation related to prostate diseases [11,12]. Prostate inflammation is typically an asymptomatic chronic inflammation characterized by histological changes and immune cell infiltration [13,14]. Chronic inflammation of the prostate may be one of the driving factors for the progression of prostate diseases, including PCa, BPH, and chronic prostatitis/chronic pelvic pain syndrome (CP/CPPS) [11,15]. Most reports indicate that chronic inflammation of prostate tissue produces free radicals for macrophage and neutrophil infiltration, inducing simple proliferation or precancerous transformation through oxidative stress on the tissue and DNA [16]. However, the source of inflammation in the prostate has not been fully elucidated; infection, autoimmune disease, cell damage, hormonal changes, and dietary factors may be responsible. Recently, the influence of the gut microbiota on human health has attracted much attention, and many studies have reported a link between the gut microbiota and various diseases, such as rheumatoid arthritis [17], gastrointestinal disorders [18], cardiovascular diseases [19], and Alzheimer's disease [20]. Although currently no evidence suggests direct effect of gut microbiota on the prostate, indirect pathological mechanisms, influence prostate health, which may be primarily related to chronic inflammation [21]. Dietary habits affect the composition of the gut microbiome. For example, a high-fat diet (HFD) leads to disruption of the intestinal flora composition, termed “gut dysbiosis,” which results in the entry of different metabolites and bacterial debris into the systemic circulation of the host, termed “leaky gut” syndrome, causing a cascade of local versus systemic inflammation, thereby affecting the host's inflammatory and immune response [[22], [23], [24], [25]]. This phenomenon also affects the regulation of prostate growth [26].

**Fig. 1:**
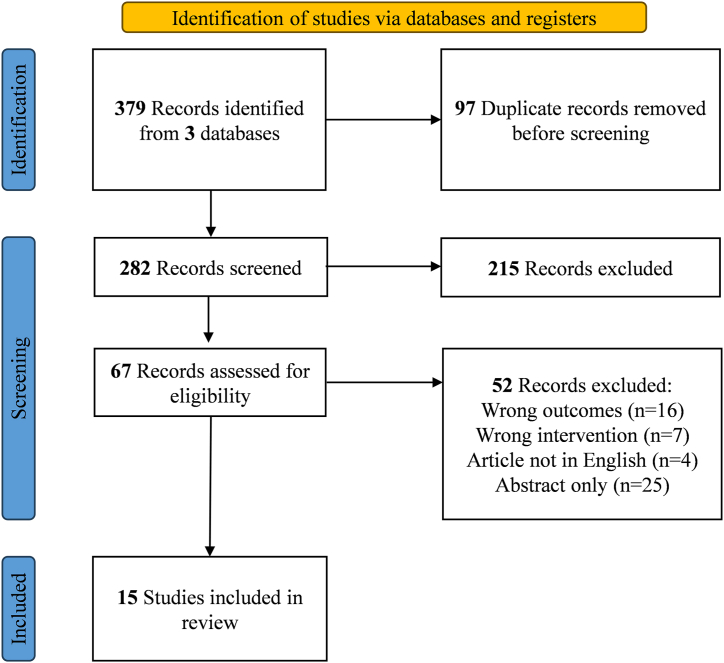
Flow chart of literature research.

To enhance the comprehensiveness of this review, we adhered to the guidelines and recommendations of the Preferred Reporting Items for Systematic Reviews and Meta-Analyses (PRISMA) (see the checklist in the supplementary material). A narrative review was conducted by searching the PubMed, Web of science, and Embase databases for publications from inception to April 25, 2023, using the following search terms to capture relevant studies: “benign prostatic hyperplasia” or “prostatitis” or “prostate inflammation” and “intestinal microbiota” or “gut microbiome.” Owing to the absence of randomized controlled trials (RCTs) in this field, we included as many case-control studies, retrospective clinical trials, and preclinical model studies as possible. Articles were excluded if they met any of the following criteria: (1) did not address at least one of the selected conditions (prostatitis, benign prostatic hyperplasia, or prostate inflammation), (2) were comments or letters to the editor, or (3) were published in a language other than English. The detailed screening process is illustrated in Fig. 1.

This review summarizes the current evidence that the gut microbiota induces prostate inflammation, its mechanisms, and future therapeutic implications.

## 2 The relationship between the microbiome and prostate inflammation and benign prostatic hyperplasia

The microbiome exists in multiple organs of the human body (e.g., gastrointestinal tract, respiratory tract, oral cavity, and urogenital tract), and it plays a crucial role in human health. Increasing evidence suggests that the human microbiome affects the development and progression of prostatic inflammation and BPH [27]. A study published in 2019 showed that men with periodontal disease had an increased risk of developing BPH and showed a clear positive association [28]. Estemalik et al. [29] found more than one oral pathogen in the prostatic secretions of patients with prostatitis and BPH, suggesting that the microbiome in the oral cavity has the potential to influence prostate health. To investigate the links between the microbiome in urine, semen, and expressed prostatic secretion (EPS) and prostate disease, numerous studies have focused on cultures of initial stream urine (VB1) and midstream urine (VB2), postprostate massage urine (VB3), semen, and EPS samples. Ivanov et al. [30] performed a bacteriological study on 48 healthy individuals and 60 patients with chronic prostatitis syndrome and reported that *Staphylococcus saprophyticus, S. epidermidis, Corynebacterium equi, C. seminale, and C. xerosis* were detected in the semen of patients with prostatitis but not in the semen of healthy men. Mandar et al. [31] applied next-generation sequencing to analyze semen samples and found that lactobacilli counts decreased and *Proteobacteria* counts increased in the semen of patients with prostatitis compared to that of healthy men. The microbiome was significantly different in the VB1 specimen cultures from those of CP/CPPS and control participants, and *Burkholderia cenocepacia* was overrepresented in CP/CPPS cases [32]. Shoskes et al. [33] analyzed urine samples using MiSeq sequencing and found that the urinary microbiome of patients with CP/CPPS had remarkably higher alpha diversity. In addition, patients with CP/CPPS had higher count of *Clostridia* (class) and lower count of *Bacilli* (class) than those in the control group. Human microbiome affects the prostate health, and development of corresponding treatment plans for prostate diseases based on alterations in the microbiome of urine, semen, and prostate secretions is possible in the future. To date, many reports have shown strong links between gut microbiota and several diseases. Based on these connections, views have been proposed for the bladder-gut-brain axis, gut-bladder axis, gut-vagina-bladder axis, and gut-kidney axis [34]. Recently, some reports have indicated the existence of the “gut-prostate axis.” [35] Table 1 lists recent studies on the association between alterations in the intestinal microbiome and prostate diseases (e.g., prostate inflammation and BPH) [[36], [37], [38], [39], [40], [41], [42]]. Golombos et al. [43] used computational genomic analysis to evaluate the intestinal microbiome of patients with prostate cancer and showed that the relative abundance of *Bacteroides massiliensis* in PCa cases was significantly increased. Konkol et al. [37] measured the gut microbiome composition of rats with chronic prostatitis using 16S rRNA gene sequencing and showed that the populations of *Rikenellaceae*, *Odoribacter*, *Clostridiaceae*, *Allobaculum*, and *Peptocaceae* were significantly higher in rats with chronic prostate inflammation (CPI) than in healthy mice. In contrast, rats with CPI showed decreased counts of *Lachnospiraceae*, *Lactobacillus*, and *Bacteroides uniformis*. Moreover, another study found that patients with CP/CPPS had significantly lower gut microbiome alpha diversity and a significantly lower count of *Prevotella*, which is sufficient as a potential biomarker [36,37]. Takezawa et al. [40] analyzed and compared the gut microbiomes of patients with enlarged prostates using 16S rRNA metagenomic analysis. These results suggest that the Firmicutes/bacteria (F/B) ratio was higher in patients with enlarged prostates than in those without. Currently, there is little research on alterations in the gut microbiome and its metabolites related to prostate inflammation; however, the above diseases mostly involve prostate inflammation. Therefore, patients with prostate inflammation may exhibit changes in the gut microbiome. However, the mechanism by which gut microbiota affect prostate inflammation is still unclear, which is an area of the future research.

**Table 1:** Relationship between alterations of intestinal microbiome and prostate inflammation, and benign prostatic hyperplasia.

Author, year of publication	Material	Sample size	Methods Aassays	Main outcomes	Reference
Prostate inflammation					
Shoskes et al. (2016)	Fecal DNA	25 patients with CP/CPPS and 25 controls	MiSeq sequencing of bacterial speciﬁc 16S rRNA capture	Patients with CP/CPPS had significantly lower gut microbiome alpha diversity and a significantly lower count of *Prevotella*, with separation sufficient to serve as a potential biomarker.	[36]
Konkol et al. (2019)	Fecal DNA	21 rats with prostate inflammation and 9 controls	16S rRNA sequencing	Rats with prostate inflammation had increased levels of *Rickenellaceae, Odoribacter, Crostridiaceae, Allobaculum* and *Peptococcaceae.* Rats with prostate inflammation had reduced levels of *Bacteroides uniformis*, *Lactobacillus,* and *Lachnospiraceae.*	[37]
Liu et al. (2021)	Cecum content	6 rats with CNP and 6 controls	16S rDNA sequences Linear discriminant analysis effect size (LEfSe)	In CNP rats, *Muribaculum* decreased and uncultured bacterium *f Desulfovibrionaceae* increased. These bacteria might serve as diagnostic markers for CNP.	[38]

Du et al. (2022)	Fecal DNA	10 EAP mice and 10 controls	16S rRNA sequencing	There were significant differences in the composition of gut microbiota between the control and EAP mice. The relative abundance of *Gemmatimonadetes, Nitrospirae,* Firmicutes, *and Fusobacteria* were lower in EAP mice. In contrast, increases in *Patescibacteria, Cyanobacteria*, and *Bacteroidetes* were detected in the EAP mice.	[39]
Benign prostatic hyperplasia					
Takezawa et al. (2021)	Fecal DNA	66 patients with BPH and 62 controls	16S rRNA sequencing	The *Firmicutes/Bacteroidetes* (F/B) ratio in the prostate enlargement group was higher than in the non prostate enlargement group (p = 0.015)	[40]
Li et al. (2022)	Fecal DNA	5 rats with BPH and 5 controls	16S rDNA sequencing	Gut microbiota beta-diversity increased (P < 0.01) in the BPH group. *Muribaculaceae* (P < 0.01), *Turicibacteraceae* (P < 0.05), Turicibacter (P < 0.01) and Coprococcus (P < 0.01) were decreased in the BPH group, whereas that of Mollicutes (P < 0.05) and *Prevotella* (P < 0.05) were increased compared with the control group.	[41]

An et al. (2023)	Fecal DNA	7 BPH, 7 BPH and finasteride treatment, and 7 controls	Linear discriminant analysis effect size (LEfSe) Kruskal–Wallis and Wilcoxon tests	*The abundance of Oscillibacter, Acetatifactor*, and *Flavonifractor* were increased in the BPH group;	[42]

Abbreviations: CP/CPPS, chronic prostatitis/chronic pelvic pain syndrome; BPH, benign prostatic hyperplasia; EAP, experimental autoimmune prostatitis; CNP, chronic nonbacterial prostatitis.

## 3 Gut microbiota inducing prostate inflammation

### 3.1 The association between intestinal permeability and prostate inflammation

Intestinal permeability is mainly determined by the intestinal barrier, which consists of three layers: the internal layer, the gut epithelial layer, and the mucus layer, which forms the intestinal immune system [44]. The mucus layer is composed of water and mucins secreted by goblet cells, which are used to separate intestinal the epithelial cells from the contents of the intestinal cavity [45]. The gut epithelial layer is formed by the connection of different types of epithelial cell junctions, mainly via tight junction proteins, adhesive band proteins, gap junction proteins and desmosomes, and is a critical part of the intestinal barrier. Tight junction proteins are composed of approximately 50 types, mainly zonula occludens (ZO), occludin (OCLN), and claudin (CLDN) [44]. The gut epithelial layer is a selective barrier primarily responsible for nutrient absorption and prevention of the entry of harmful substances. Additionally, certain microorganisms in the intestine produce substances that are beneficial to the intestinal barrier, such as Short-chain fatty acids (SCFAs). Zhang et al. [46] showed that dietary butyrate (a type of SCFA) can upregulate the expression of genes related to the ileal epithelial barrier, claudin-1, claudin-2, occludin, junction adhesion molecule 3 (JAM 3), and zonula occludens-1 (ZO-1), to maintain the integrity of the intestinal barrier. In contrast, Yang et al. [47] found that feeding an HFD caused “gut dysbiosis” in mice, leading to a significant decrease in the expression of tight junction proteins in the colonic epithelial cell layer. When the gut microbiota is disrupted or dysregulated, gut barrier function may be destroyed, which increases intestinal permeability and causes “leak gut syndrome”. The gut microbiota and their metabolites are translocated from the intestine, activating immunity and leading to the release of inflammatory and pro-inflammatory factors [48]. If gut microbiota metabolites (such as SCFAs) or bacterial components (such as lipopolysaccharides) enter the systemic circulation through the venous and lymphatic pathways, they may even lead to systemic inflammatory reactions and cause prostate inflammation [22,49].

### 3.2 SCFAs and prostate inflammation

The gut microbiota can produce biologically active metabolites, such as bile acid derivatives, SCFAs, and tryptophan metabolites, which participate in various mechanisms and pathways that affect the physiological and pathological processes of the host [50]. SCFAs are among the most important metabolites of the gut microbiota. They are produced by the decomposition of the gut microbiome and the fermentation of dietary fibers. They include, acetate, propionate and butyrate. In *Firmicutes*, acetate and propionate are mainly produced by *Bacteroides*, whereas butyrate is mainly produced by *Roseburia intestinalis* in *Firmicutes* [51,52]. SCFAs are crucial for the regulation of the immune system, metabolism, and cell proliferation [53,54]. In recent years, many studies have shown that SCFAs are associated with urinary system diseases, such as PCa [55], bladder cancer [56], and benign prostatic hyperplasia [49]. SCFAs are recognized by G protein-coupled receptors (GPCRs); GPR43, GPR41, PLFR78, and GPR109A have been identified as SCFA receptors [57]. SCFAs (such as propionate) can bind to GPR43 and regulate various immune cells, including regulatory T cells (Treg cells), Th17 cells, macrophages, and B cells, by inhibiting histone deacetylase (HDAC) [58,59], thereby inhibiting inflammation. In addition, butyrate can bind to GPR109A to regulate Tregs and induce CD4^+^ T cells to produce more interleukin-10 (IL-10) and less interleukin-17 (IL-17), thereby inhibiting colonic inflammation [60]. In contrast, butyrate binding to GPCRs also activates NLRP3 inflammasomes, causing the expression of some proinflammatory factors, such as interleukin-1 β and interleukin-18. Generally, butyrate exerts anti-inflammatory effects on the intestinal immune microenvironment [61]. Therefore, when gut dysbiosis occurs, the number of bacteria producing SCFAs decreases, and the number of harmful bacteria increases, leading to the expression of some inflammatory cells and the release of proinflammatory cytokines. In fact, the immune response induced by gut microbiota dysregulation occurs at both local and systemic levels, and the production of proinflammatory cytokines (e.g., IL-17, IL-23, TNF-α, IFN-γ) may stimulate inflammation at distant sites, including the prostate, via circulation [43]. Ratajczak et al. [49] measured the concentrations of SCFAs in rat fecal samples using gas chromatography and found that the concentrations of SCFAs such as butyric acid, valeric acid and caproic acid in the feces of rats with CPI were significantly lower than those in healthy rats, indicating that CPI is related to changes in SCFAs. Du et al. [39] measured the composition and metabolites of the gut microbiota in an experimental autoimmune prostatitis (EAP) mouse model and in a control group via 16S rRNA sequencing and found that the communities of bacteria that produce SCFAs in EAP mice were significantly reduced. On the other hand, the ratios of Th 17 and Treg in the EAP mice and the control group were also measured, and it was found that Th 17/Treg was in an imbalanced state in the EAP model. The Th17/Treg imbalance in the EAP model was corrected by supplementation with propionic acid, indicating that intestinal microbiota imbalance leads to prostate tissue inflammation through the reduction of propionic acid, and propionate supplementation could be used as a therapeutic approach to alleviate prostate inflammation in EAP mice [39].

### 3.3 Microbial-associated molecular patterns

**Fig. 2:**
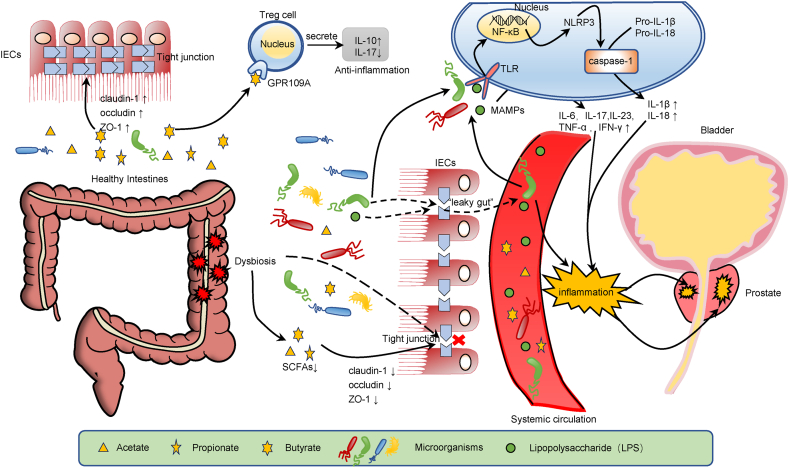
Schematic diagram of prostatic inflammation caused by gut dysbiosis. The diagram is divided into two parts: 1) The intestinal tract of healthy people is in a dynamic balance, and the metabolites derived from intestinal microorganisms, butyrate, can upregulate the expression of claudin-1, occludin, and zonula occludens 1 (ZO-1) to enhance tight junctions and protect intestinal barrier function. In addition, butyrate binds to GPR109A and regulates the secretion of IL-10 and IL-17 by Treg cells to inhibit inflammation. 2) When “gut dysbiosis” occurs, the number of microorganisms producing SCFAs in the gut decreases, and the number of harmful bacteria increases, resulting in damage to the intestinal barrier and “leaky gut” syndrome, which leads to the translocation of bacteria and bacterial component lipopolysaccharide and causes systemic inflammation. Bacteria and LPS bind to TLRs as MAMPs, upregulate the expression of NF-κB, and activate the NLRP3 inflammasome. Activated NLRP3 activates caspase-1, and activated caspase-1 promotes the maturation of pro-IL-1β and pro-IL-18 to IL-1β and IL-18. In addition, LPS can also bind to other PRRs and activate innate immune cells to secrete IL-17, IL-23, TNF-α, and IFN-γ to mediate the occurrence of inflammation. Finally, LPS and various inflammatory factors travel through the circulation to the prostate and cause an inflammatory response in the prostate. Abbreviations: IECs, intestinal epithelial cells; ZO-1, zonula occludens 1; IL, interleukin; SCFAs, short-chain fatty acids; TLR, Toll-like receptor; NF-κB, nuclear factor-kappaB; MAMPs, microbial-associated molecular patterns; NLRP3, nucleotide-binding domain leucine-rich repeat (NLR) and pyrin domain containing receptor 3.

Inflammatory processes are initiated by binding of microbial-associated molecular patterns (MAMPs) to pattern recognition receptors (PPRs) [62]. MAMPs are also referred to as pathogen-associated molecular patterns (PAMPs), which refer to the specific structural or chemical patterns of microorganisms, such as the bacterial flagellum, peptidoglycan and lipopolysaccharide (LPS). They can bind with PPRs such as Toll-like receptor (TLR), NOD receptor (NLR), and leucine rich alpha-2-glycoprotein 1 (LRG1) to activate innate immune cells [63,64], activating innate immune cells and inducing the secretion of proinflammatory cytokines (e.g., interleukin-17, interleukin-23, TNF-α, and IFN-γ) [65]. When gut dysbiosis occurs, the intestinal barrier is disrupted, tight junction protein function is impaired, and intestinal permeability is increased, allowing the translocation of bacteria and microbial-derived products, leading to the development of MAMP-mediated inflammatory responses. Therefore, LPS might play a crucial role in this process [1]. As a PAMP, LPS can upregulate the expression of nuclear factor-kappa B (NF-κB) and Pro-IL-1β by binding to TLR. The former activates the NLRP3 inflammasome complex (also a PRR), and the activated NLRP3 inflammasome activates caspase-1. Active caspase-1 promotes the maturation and secretion of pro-IL-1β and pro-IL-18 [66]. Dos Santos Gomes et al. [67] found that the transurethral injection of LPS to stimulate the expression of proinflammatory cytokines is an effective method for establishing a model of prostatitis. In another study, LPS induced prostatitis by binding to TLR4, inducing the upregulation of nuclear factor-kappaB (NF-κB) expression and the release of inflammatory cytokines, and LPS-induced prostatitis also increased prostate cancer metastasis [68]. Zhong et al. [69] used broad-spectrum antibiotics to cause intestinal microbial dysbiosis in mice, which manifested as a significant increase in the relative abundance of *Proteobacteria,* and significantly increased LPS levels in the serum and tumors. LPS stimulated the secretion of inflammatory cytokines (mainly IL-6) through the TLR4-NF-κB pathway to promote prostate inflammation. In addition, intratumoral LPS was elevated and promoted the progression of PCa in mice through the NF-κB-IL 6-STAT 3 axis. Cani et al. [70] found that a high-fat diet (HFD) increased LPS-containing gut bacteria in mice. HFD causes gut dysbiosis, reduces the alpha diversity of the intestinal flora, decreases the level of ZO-1 in the intestinal epithelial barrier, increases intestinal permeability, and allows LPS to enter the circulatory system [71]. In addition, LPS entering the circulation may upregulate the expression of HDC, the histamine biosynthesis gene, in the prostate tissue; mast cells and other immune cells are also activated by LPS, thereby inducing a local inflammatory response in the prostate [72]. Moreover, TAK-242, an inhibitor of TLR4 that recognizes LPS, inhibited mast cell activation in a mouse model of prostate cancer, resulting in a significant decrease in the proportion of degranulated mast cells in tumors [72]. Fig. 2 summarizes several pathological mechanisms of prostate inflammation induced by the gut microbiome. These results suggest that TAK-242 inhibits prostate tumor growth by inhibiting mast cell activity, which could be a useful research direction for the future.

In summary, LPS from certain gut bacteria can induce immune responses through multiple pathways and lead to inflammation under conditions of gut microbiota imbalance. Moreover, this inflammatory response is elicited not only locally but also in the peripheral circulation, causing systemic inflammation, which may reach the prostate and cause local inflammation [49].

## 4 Manipulation of gut microbiota and prostate inflammation

**Fig. 3:**
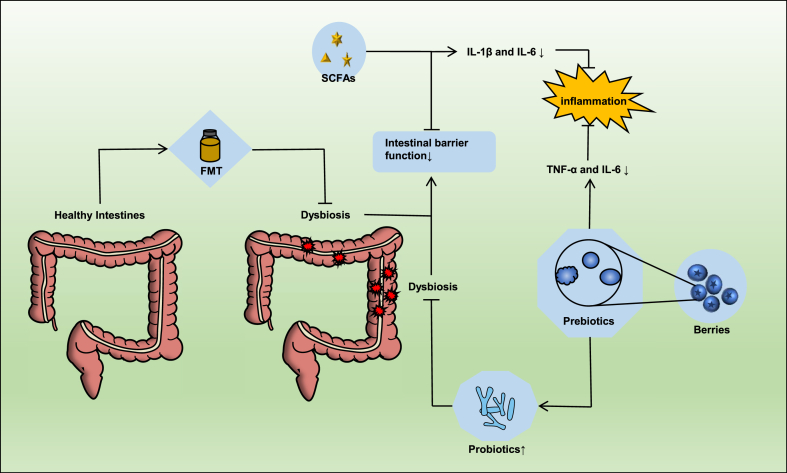
Prebiotics significantly reduced the serum levels of TNF-α and IL-6 in mice and alleviated systemic inflammation. On the other hand, probiotics are fermented by beneficial bacteria in the gut and stimulate the growth of probiotics in the intestine. Supplementation with probiotics can regulate the composition of the gut microbiota to correct intestinal flora imbalance. FMT is the transplantation of intestinal microorganisms from healthy donors to patients to regulate intestinal flora imbalance. Dietary SCFAS supplementation significantly altered the composition of the intestinal flora, protected intestinal barrier function, and reduced the levels of IL-1β and IL-6 in the ileum to inhibit the inflammatory response in rabbits. Abbreviations: FMT, fecal microbial transplant; SCFAs, short-chain fatty acids; IL, interleukin; TNF-α, tumor necrosis factor-α.

It is well known that the use of multimodal treatment regimens is more effective in controlling prostatic inflammation than monotherapy. Antibiotics are the first line of treatment for bacterial prostatitis, but antibiotics alone may not resolve patients with urinary tract symptoms. For example, a multimodal treatment regimen of alpha blockers and nonsteroidal anti-inflammatory drugs (NSAIDS) for chronic prostatitis with pain can significantly alleviate symptoms and improve patient quality of life [64]. As described above, the occurrence of many diseases, including prostate inflammation, is related to an imbalance in the intestinal flora, so it is particularly crucial to maintain the balance of the gut microbiome. Intervention of the intestinal flora to change gut dysbiosis and protect the intestinal barrier may prevent or reduce the risk of prostatitis. The main interventions included dietary modification or SCFAS supplementation, probiotics, prebiotics, and fecal microbiota transplantation (Fig. 3).

### 4.1 Probiotics

There are many microbiomes in the human body. They are widely distributed in the mouth, skin, respiratory tract, urinary tract and gastrointestinal tract. The microbiome is a symbiotic relationship with the human body that has key effects on maintaining human health. The microbiome is generally divided into three groups, probiotics, harmful bacteria and neutral bacteria, and in a healthy human body, these three bacteria are always in dynamic balance. Probiotics can effectively modulate intestinal inflammation by changing the composition, metabolism, and functional properties of the innate gut microbiota [73]. Harmful bacteria will not cause disease to the host under normal circumstances, but if the intestinal flora is dysregulated, the number of harmful bacteria increases beyond a certain range, which will cause disease to the host. Therefore, intervention in the intestinal flora is promising as an adjuvant therapy for inflammation and cancer in the future. For example, in colon cancer patients, the abundance of butyrate-producing bacteria is increased after the administration of probiotics, which helps to maintain intestinal barrier function to avoid the activation of inflammation-related factors in the tumor microenvironment [74,75]. Henrick et al. [76] found that the levels of inflammatory factors such as interleukin-13, interleukin-17A, interleukin-21, and interleukin-31 decreased in infants fed *Bifidobacterium infantis EVC001*, indicating that feeding infants *Bifidobacterium infantis EVC001* can suppress early intestinal inflammation. In a recent systematic review that included nine studies to evaluate the effect of probiotics on urinary tract infections, two of them concluded that probiotics significantly reduce the risk of recurrent urinary tract infections [77]. Probiotics as a food supplement may have an adjuvant effect on the treatment of inflammation and cancer, but their safety has not been completely solved, which is worthy of further investigation in future research.

### 4.2 Prebiotics

Prebiotics are nondigestible food components, including polyphenols, polysaccharides, protein hydrolysates, oligosaccharides, etc. They can be fermented by beneficial bacteria in the gut to produce bioactive products that are beneficial to host health and stimulate the growth of probiotics in the gut [78]. Berries are rich in polyphenols such as quercetin, kaempferol, ellagin and anthocyanins. These compounds generally possess antioxidant and anti-inflammatory effects, positively regulate the gut microbiota and have therapeutic effects on gut-related inflammation, cancers, and metabolic disorders [79]. The serum levels of TNF-α and IL-6 were significantly decreased in mice supplemented with grape polyphenols, indicating that the feeding of grape polyphenols can reduce systemic inflammation. In addition, as mentioned above, HFD can lead to the impairment of intestinal barrier function, causing the translocation of bacteria and their derivatives (LPS), resulting in an increase in serum LPS levels. However, feeding mice with grape polyphenols can counteract the HFD-induced gastrointestinal effects and reduce the serum LPS level of mice [80]. Choe et al. [81] found that the extract of blackberry seed flour could regulate intestinal microbiota and inhibit LPS-induced IL-1β production, but the specific mechanism is still unclear, and further studies are needed. A recent study showed that quercetin could reduce the secretion of the inflammatory chemokine CCL2 by inhibiting the MAPK, NF-kB and STAT3 pathways, thereby alleviating the symptoms of CP/CPPS and enhancing the resolution of inflammation [82]. In addition, Yu et al. [83] found that the metabolite of poria cocos polysaccharide (PPs-FM) could have an anti-inflammatory effect on relieving chronic nonbacterial prostatitis by promoting the increase in probiotics (e.g., *Parabacteroides* and *Parasutterella*) and beneficial metabolites (e.g., a free bile acid: 7-ketodeoxycholic acid). Chen et al. [84] demonstrated that long-term oral administration of glycated whey proteins can significantly reduce the number of macrophages, CD8^+^ T cells and B cells to control and slow the inflammatory progression of autoimmune prostatitis.

### 4.3 Fecal microbiota transplantation (FMT)

Fecal microbiota transplantation refers to the transplantation of intestinal microorganisms from healthy donors to patients through upper or lower gastrointestinal routes to restore the homeostasis of the intestinal microbiota [85]. Currently, FMT is approved for the treatment of recurrent *Clostridium difficile* infections, and it can prevent 90 % of recurrent *Clostridium difficile* infections [86]. In a recent study, Du et al. [39] transplanted fecal suspensions from EAP mice into germ-free mice treated with antibiotics, resulting in a significant reduction in the proportion of Treg cells in germ-free mice, indicating that fecal microbes in EAP mice are associated with autoimmune prostatitis. However, Du et al. did not attempt to transplant fecal suspensions from healthy mice into EAP mice to investigate whether this approach could improve symptoms of prostatitis. In addition, Tariq et al. [87] showed that FMT reduced the colonization of multidrug-resistant *E. coli* and reduced the frequency of recurrent urinary tract infections. Intervention therapy with FMT has promising prospects, but its safety remains controversial due to unknown fecal bacterial composition and pathogenicity.

### 4.4 Dietary supplementation

The composition of the gut microbiota is related to age, sex, dietary habits, lifestyle, and other factors. In particular, dietary habits can directly affect the health of the gut microbiota and affect host inflammation and even cancer progression. For example, the “Western dietary pattern” is the feature of red meat, potatoes, and high-fat dairy consumption being higher, and this diet increases the risk of PCa. In contrast, the characteristics of the “prudent dietary pattern” are to eat more vegetables, fruits, legumes, and fish, and this kind of diet reduces the incidence of PCa [24]. In addition, the incidence of inflammatory diseases and cancer increases when diets low in SCFAs are continuously consumed or when SCFAs are reduced in feces [55]. Du et al. [39] concluded that dietary supplementation with propionate significantly ameliorated prostatitis and pelvic pain in EAP mice. Zhang et al. [46] found that dietary butyrate supplementation significantly changed the composition of intestinal microbes and reduced the levels of IL-1β and IL-6 in the ileum of rabbits, suggesting that dietary butyrate supplementation helps to protect intestinal barrier function and inhibit inflammation. Additionally, it should be considered that recently introduced compounds have been demonstrated having a significant influence on microbiota. The use of postbiotics [88], lysates [89] and Paraprobiotics [90] can modify oral microbiota so also these products should be considered in future research, as adjuvants, also for gut microbiota management and prostate illness prevention. Therefore, dietary interventions may be a useful approach to prevent or delay the development of prostatic inflammation or even prostate cancer.

This review also has some limitations. First, the gut microbiota consists of a large number of diverse bacteria, and there may be synergistic or antagonistic interactions between different bacterial species. This variability contributes to significant differences across the studies analyzed in this review. Second, regarding the therapeutic aspects, the outcome variables were not consistent across all studies, which limits this review to summarizing potential methods that could be applied in the future to prevent or delay the development of prostate inflammation or BPH. Third, we applied strict criteria during the literature selection process, and it is possible that relevant studies were not comprehensively retrieved. This approach may have further limited the number of studies included and potentially result in an incomplete reflection of the mechanisms by which gut microbiota influence prostate inflammation and BPH.

## 5 Risk of bias

**Fig. 4:**
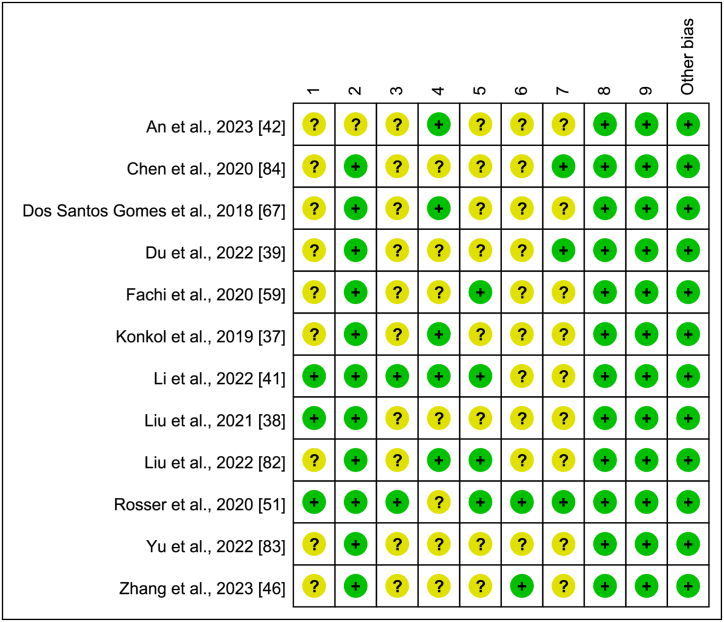
SYRCLE Risk of Bias for animal studies.

**Fig. 5:**
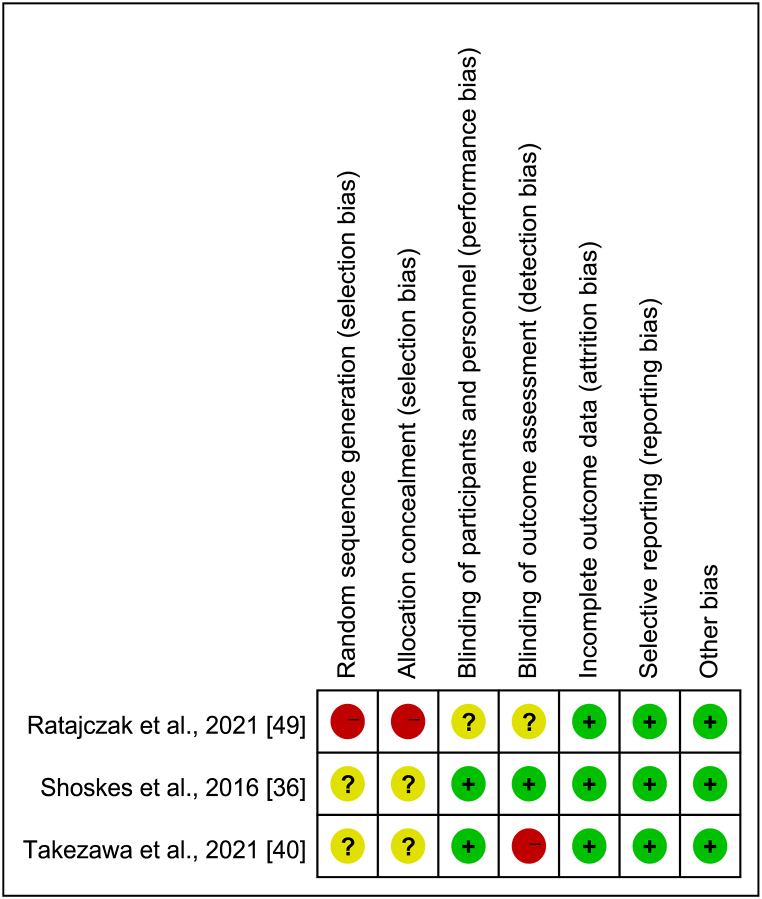
Cochrane risk of bias for human studies.

For the included animal studies, we assessed the risk of bias using the SYRCLE (Systematic Review Centre for Laboratory Animal Experimentation) Risk of Bias tool [91] (Fig. 4). For the included human studies, we used the Cochrane Risk of Bias tool (Fig. 5). A green symbol indicates a low risk of bias, a yellow symbol indicates a moderate risk of bias (or insufficient information), and a red symbol indicates a high risk of bias.

The risk of bias was unclear in most animal studies. Nearly all studies did not report random outcome assessment, allocation concealment, baseline characteristics, blinding of personnel, or random housing. Of the 12 animal studies, 3 reported using a sequence generation method, 2 reported random housing, and 2 reported random selection of animals for outcome assessment.

For the human studies, all three studies had a high overall risk of bias. Since none were randomized controlled trials, none clearly used random sequence generation. Among these studies, one was double-blinded, and one study implemented blinding of outcome assessors. All three studies were deemed to have a low risk of bias for incomplete outcome data.

SYRCLE's RoB tool was used to address the following domains: 1, Was the allocation sequence adequately generated and applied? 2, Were the groups similar at baseline, or were they adjusted for confounders in the analysis? 3, Was the allocation to the different groups adequately concealed? 4, Were the animals randomly housed during the experiment? 5, Were the caregivers and/or investigators blinded from knowledge of which intervention each animal received during the experiment? 6, Were animals selected at random for outcome assessment? 7, Was the outcome assessor-blinded? 8, Were incomplete outcome data adequately addressed? 9, Are reports of the study free of selective outcome reporting?

## 6 Conclusions

The gut microbiota plays an important role in maintaining the health of the host. In recent years, a large number of studies have suggested that the gut microbiome may participate in the progression of prostatitis, BPH and PCa through different mechanisms, proving the existence of the gut-prostate axis [35,38,40,92]. This review mainly focused on the relationship between gut microbiota and prostate inflammation and benign prostatic hyperplasia. At present, the mechanisms by which the gut microbiota induces inflammation of the prostate may be as follows: 1) Dysbiosis of the gut microbiota reduces the number of SCFA-producing probiotics and increases the number of harmful bacteria, which weakens the anti-inflammatory effect of SCFAs. 2) Disruption of the integrity of the gut barrier leads to translocation of bacteria and bacterial component substances such as LPS, which mediates prostatic inflammation through microbiota-associated molecular patterns. In addition, based on the existing mechanism, some intervention measures for gut microbiota, such as probiotics, prebiotics, FMT and SCFA dietary supplements, have promising prospects. If these treatment measures still have some potential safety risks, further studies are needed. Nevertheless, the current evidence about the relationship between the gut microbiome and prostate inflammation is only the tip of the iceberg. Further studies are needed to investigate the role and specific mechanisms of the gut microbiota in the progression of prostatic inflammation, which will help to develop potential new therapeutic targets for prostate inflammation and benign prostatic hyperplasia.

## Funding

This study was funded by the 10.13039/501100001809National Natural Science Foundation of China (Grant Number 82000721) and Program from the 10.13039/501100004829Department of Science and Technology of Sichuan Province (Grant Number 22NSFSC1750).

## Data availability statement

No data was used for the research described in the article.

## CRediT authorship contribution statement

**Jie Chen:** Writing – original draft, Investigation. **Bo Chen:** Writing – original draft, Investigation. **Bin Lin:** Writing – original draft, Investigation. **Yin Huang:** Writing – review & editing. **Jinze Li:** Writing – review & editing. **Jin Li:** Writing – review & editing. **Zeyu Chen:** Writing – review & editing. **Puze Wang:** Resources. **Biao Ran:** Resources. **Jiahao Yang:** Resources. **Huijian Huang:** Resources. **Liangren Liu:** Writing – review & editing, Supervision. **Qiang Wei:** Writing – review & editing, Supervision. **Jianzhong Ai:** Writing – review & editing, Supervision, Conceptualization. **Dehong Cao:** Writing – review & editing, Validation, Funding acquisition, Conceptualization.

## Declaration of competing interest

The authors declare that they have no known competing financial interests or personal relationships that could have appeared to influence the work reported in this paper.
